# 间充质基质细胞治疗激素耐药急性移植物抗宿主病的机制与临床研究进展

**DOI:** 10.3760/cma.j.cn121090-20250521-00240

**Published:** 2026-02

**Authors:** 楠 王, 郑丽 徐, 代红 刘

**Affiliations:** 1 中国人民解放军总医院血液病医学部、血液与健康全国重点实验室，北京 100853 Senior Department of Hematology, Chinese PLA General Hospital, State Key Laboratory of Experimental Hematology, Beijing 100853, China; 2 北京大学人民医院、北京大学血液病研究所、国家血液系统疾病临床医学研究中心，北京 100044 Peking University People's Hospital, Peking University Institute of Hematology, National Clinical Research Center for Hematologic Disease, Beijing 100044, China

## Abstract

急性移植物抗宿主病（aGVHD）是异基因造血干细胞移植后的主要并发症。糖皮质激素作为标准一线治疗药物的疗效有限，超过40％的耐药患者预后不佳。近年来，间充质基质细胞（MSC）作为激素耐药aGVHD的二线治疗药物在国内外被广泛研究。2025年国内首个MSC产品获国家药品监督管理局批准附条件上市，用于治疗14岁以上激素耐药的aGVHD患者。基于此，本文对MSC治疗激素耐药aGVHD的临床研究进展进行综述，重点探讨其作用机制与疗效评价，旨在加深对MSC临床潜力和现存挑战的理解。

## Abstract (en)

Acute graft-versus-host disease（aGVHD）is a common complication of allogeneic hematopoietic stem cell transplantation. The efficacy of standard first-line glucocorticoid treatment is limited; more than 40％ of patients develop steroid-refractory aGVHD with an extremely poor prognosis. In recent years, mesenchymal stromal cell（MSC）as a second-line treatment for steroid-refractory aGVHD has been widely studied worldwide. In 2025, the first domestic MSC product was conditional approved by the National Medical Products Administration for steroid-refractory aGVHD in patients aged 14 years and above in China. In light of this, this article reviews the clinical research progress of MSC in the treatment of steroid-refractory aGVHD, focusing on mechanisms and efficacy evaluation, aiming to deepen the understanding of the clinical potential and existing challenges of MSC.

异基因造血干细胞移植（allo-HSCT）是许多恶性、重症血液病的有效治疗手段，甚至是唯一的治愈方式，而急性移植物抗宿主病（aGVHD）是allo-HSCT的常见并发症和主要死亡原因[1]。aGVHD的一线治疗药物是糖皮质激素，然而，约40％的Ⅱ度和60％～70％的Ⅲ/Ⅳ度aGVHD患者对其耐药[2]–[3]。传统的二线治疗药物集中于更广泛、强效的免疫抑制剂，但其增加致死性感染的发生风险。因此，探索强免疫抑制作用之外的二线治疗方法刻不容缓。间充质基质细胞（MSC）具有免疫调节、抗炎和促进免疫损伤的组织修复再生的作用。近年来，Temcell（骨髓MSC产品）于2015年在日本被批准用于治疗儿童和成人aGVHD[4]；Remestemcel-L（骨髓MSC产品）于2024年12月在美国被批准用于治疗激素治疗失败的儿童aGVHD[5]。人脐带来源的MSC于2025年1月在中国被附条件批准用于14岁以上患者治疗激素治疗失败的aGVHD[6]。本文系统梳理MSC治疗激素耐药aGVHD的临床研究进展，重点探讨其作用机制与疗效评价，以加深对MSC临床潜力和现存挑战的理解。

一、MSC生物学特性

MSC被定义为具有多能性和贴壁性的成纤维细胞样基质细胞群，其特征是具有自我更新能力以及向脂肪细胞、成软骨细胞和成骨细胞多谱系分化的潜能[7]。MSC最初在骨髓中被发现，由美国Arnold Caplan教授在1991年首次命名为“间充质干细胞”，他发现MSC具有免疫调节、抑制细胞凋亡和疤痕形成、刺激血管再生以及促进组织干细胞生长的能力。后续研究发现MSC同样存在于脂肪组织、脐带、脐血、牙髓、胎盘、滑膜等组织和部位中，并为邻近器官特异性细胞提供结构和营养支持[8]。由于组织来源及细胞分离培养方法的差异，MSC表型具有异质性且缺乏特异性表型标志物。2006年，国际细胞治疗学会（ISCT）提出MSC的最低定义标准[9]：具有黏附于塑料贴壁生长特性，MSC免疫表型特征以及体外分化为成骨、成软骨、成脂肪细胞的潜能。其中，免疫表型定义为表达MSC标志物CD73、CD90和CD105，同时缺乏单核细胞和巨噬细胞标志物（CD11b或CD14）、造血祖细胞和内皮细胞标志物（CD34）、白细胞标志物（CD45）、B细胞标志物（CD19或CD79a）和HLA-DR的表达。后续研究表明，只有小部分细胞可表现出真正的干细胞特性，而大多数细胞为不同组织中分离出的异质性基质细胞群[10]。因此，2025年ISCT更新共识，指出使用“间充质基质细胞”作为标准用语[11]。

研究发现不同组织来源的MSC在转录组、蛋白质组、免疫表型和免疫调节功能方面存在差异，表明MSC具有不同方向的分化潜能[12]。骨髓来源的MSC具有较强的成骨和成软骨能力；而滑膜来源的MSC则表现出较强的增殖能力和成软骨潜能；脐带来源的MSC与其他成人来源的MSC相比，具有更多生物学优势，如更易获取、扩增规模更大、分化和增殖能力更强[7]。Wang等[13]通过单细胞测序技术检测来自于骨髓、脂肪、脐带和真皮的不同传代次数的MSC，依据特征性基因表达鉴定出7个组织特异性和5个保守的MSC亚群并提示脐带特异性亚群在免疫抑制特性方面具有优势。

二、MSC发挥免疫调节、治疗aGVHD的作用机制

MSC对先天性和适应性免疫细胞均具有免疫调节作用[8]。可通过直接的细胞-细胞接触、生物活性因子的分泌和（或）两种机制的组合作用于T细胞、B细胞、树突状细胞（DC）、自然杀伤细胞、巨噬细胞等，从而行使免疫调节、再生和修复功能[14]。MSC的旁分泌效应依赖于环境刺激。当受到外界刺激时，MSC向周围环境中释放特定介质包括细胞外囊泡（EV）、生长因子、细胞因子、趋化因子等[14]。大多数分子作为旁分泌因子来调节靶细胞的细胞内信号通路，其中，白细胞介素-6（IL-6）、IL-10、转化生长因子-β1（TGF-β1）、肿瘤坏死因子-α（TNF-α）、前列腺素E2（PGE2）、肝细胞生长因子（HGF）、成纤维细胞生长因子、吲哚胺-吡咯2,3-双加氧酶（IDO）以及一氧化氮被证明可以调节免疫反应，抑制细胞死亡和纤维化，刺激血管形成与组织重塑，并促进伤口愈合[15]。

（一）MSC治疗aGVHD的作用机制

MSC在aGVHD中的研究可追溯到2002年，Di Nicola等[16]首次发现MSC可降低造血干细胞移植后重度GVHD发生率。2004年，Le Blanc等[17]首次应用第三方骨髓来源的MSC成功治疗难治性Ⅳ度、同时累及肠道和肝脏的aGVHD。MSC治疗aGVHD作用机制包括以下3个方面[18]。

1. 旁分泌作用：分泌多种细胞因子，抑制促炎性免疫细胞的增殖和功能，增加抗炎性免疫细胞的数量，减轻组织损伤，促进组织修复。MSC可通过诱导IL-10、PGE2产生，抑制IL-17、IL-22和γ干扰素（IFN-γ）分泌，从而抑制辅助性T细胞17（Th17）的分化[19]。体内外敲低IL-25的实验结果表明，MSC可以通过调节IL-25/信号转导及转录激活因子3（STAT3）/程序性细胞死亡蛋白配体1（PD-L1）轴来抑制Th17生成[20]。此外，MSC分泌的IDO可导致局部微环境中色氨酸浓度降低及代谢产物积累，抑制效应T细胞活化，同时促进叉头样转录因子3表达及调节性T细胞（Treg）分化，从而诱导免疫耐受[21]。研究表明脂多糖（LPS）或TNF-α激活的骨髓来源MSC可以通过释放PGE2对巨噬细胞进行重编程，PGE2通过结合巨噬细胞受体EP2和EP4受体，激活环磷酸腺苷-蛋白激酶A通路，诱导IL-10表达，促进其向M2型巨噬细胞抗炎表型转化[22]。在MSC衍生的EV中富集的微小RNA（miR-21-5p）已被证明会影响DC的成熟和功能以及促进抗炎型M2巨噬细胞极化[23]。此外，EV还能够转移表观遗传调控因子，在局部和全身微环境中协调细胞间的表观遗传重编程和信号调节[24]。

2. 细胞-细胞接触作用：MSC可通过与各类免疫细胞表面分子和受体直接作用来调节免疫细胞的下游信号通路，从而影响T细胞活化、增殖和功能。在体外研究中，MSC通过上调细胞间黏附分子-1和血管细胞黏附分子-1来抑制初始和记忆T细胞与抗原呈递细胞间通讯[25]。MSC还可以通过程序性细胞死亡蛋白1（PD-1）/PD-L1通路防止供体T细胞过度活化[26]。此外，有研究报道MSC可通过转移其活性线粒体与质膜片段，直接与Treg细胞进行通讯。并且，这一转移事件具有HLA依赖性，其效率与Treg和MSC供体之间的HLA-C及HLA-DRB1表位错配负荷相关[27]。MSC还可通过向CD8^+^T细胞转移线粒体，下调后者向细胞毒性T细胞转化的关键转录因子表达，从而抑制CD8^+^T细胞的扩增及IFN-γ产生[28]。

3. MSC凋亡介导免疫抑制：MSC通过补体介导的细胞毒效应被杀伤性细胞攻击而迅速凋亡，随后被巨噬细胞吞噬后诱导极化，释放多种因子（IL-10、IDO及TGF-β等）抑制免疫效应细胞，从而诱导免疫耐受[29]。GVHD小鼠模型研究报道，在MSC输注后，细胞毒性细胞释放穿孔素诱导MSC凋亡，随后巨噬细胞吞噬凋亡的MSC，继而产生IDO，发挥免疫抑制作用[30]。

（二）MSC优化处理

MSC的免疫调节特性受炎症微环境影响。在强炎症环境如高浓度IFN-γ或TNF-α环境下，MSC表现出强大的免疫抑制活性；而在弱炎症或稳态环境下，MSC则表现弱免疫调节甚至起到免疫促进活性[8]。在激素耐药GVHD背景下，免疫抑制剂耐药的免疫微环境中丰富的炎症细胞因子可能促使MSC表现出强大的免疫抑制活性，从而促进组织修复[31]。研究提示对MSC进行修饰可以增强其免疫调节功能[32]。IFN-γ和TNF-α处理的脐血MSC表现出几丁质酶-3样蛋白1的表达增加，同时通过STAT3激活来抑制Th17分化[33]。细辛脂预处理的脐带MSC可显著抑制aGVHD模型小鼠的CD4^+^和CD8^+^T细胞增殖，下调Th1型细胞因子并上调Th2型细胞因子的产生，减少肝、肺和肠的炎性损伤[34]。此外，用IFN-γ和环孢素（CsA）预处理MSC也可以延长其在人源化aGVHD小鼠模型中存活时间[35]。缺氧预处理通过缺氧诱导因子-1α/趋化因子受体4（CXCR4）和CXCR7通路增加长链非编码RNA-p21的表达，从而促进MSC的迁移和存活[36]。另一项研究也表明缺氧预处理的凋亡MSC具有更强的免疫编程能力[37]。在人源化GVHD小鼠模型中，稳定表达CXCR4和IL-10的MSC相较于野生型MSC，向靶器官迁移的能力更强，免疫调节特性也更强[38]。代谢重编程也可增强MSC的治疗效果，研究证实MSC通过三磷酸腺苷合成酶抑制剂介导的单磷酸腺苷活化的蛋白激酶依赖性糖酵解重编程可增强其免疫抑制作用[39]。MSC经包括IFN-γ、IL-17、IL-1β和TNF-α在内的细胞因子处理后可增强代谢适应性、糖酵解能力和免疫抑制能力[40]。

三、MSC治疗激素耐药aGVHD的临床研究进展

（一）MSC治疗激素耐药aGVHD的整体效果

Le Blanc等[41]在激素耐药的成人和儿童aGVHD患者中开展了一项Ⅱ期、单臂、前瞻性研究，该研究结果显示在激素联合钙调蛋白抑制剂或吗替麦考酚酯（MMF）基础上加用MSC治疗6周，患者客观缓解率（ORR）达70.9％。2014年Kurtzberg等[42]报道Remestemcel-L治疗75例激素耐药aGVHD患儿28 d的ORR为61.3％，且患者耐受性良好；其中Ⅲ～Ⅳ度aGVHD患儿占88％，肠道受累患儿占86.7％，激素治疗无效后接受过≥2线治疗的患儿占60％；Remestemcel-L的单次输注剂量为2×10^6^/kg，中位输注次数为10（1～20）次，输注>8次的患儿占53.3％。2020年Kurtzberg等[43]再次报道该研究扩大样本量后的数据：241例Ⅱ～Ⅳ度激素耐药aGVHD患儿接受Remestemcel-L治疗后的第28天ORR为65.1％，且耐受性良好。同年发表的另一项Ⅲ期、前瞻性、单臂、多中心研究同样对Remestemcel-L在激素耐药aGVHD患儿中的应用进行了评估，结果显示，第28天ORR为70.4％，显著超过预先设定的45％目标值；完全缓解（CR）率从治疗第28天的29.6％上升至第100天的44.4％，研究结果支持其在儿童患者中的潜在疗效[44]。Nagamura-Inoue等[45]报道了脐带来源MSC（IMSUT-CORD）治疗成人激素耐药aGVHD的Ⅰ期剂量递增研究结果，研究共纳入7例患者，其中4例单次输注剂量为1×10^6^/kg、3例为2×10^6^/kg，中位输注次数为7（4～8）次；在首剂IMSUT-CORD输注16周后，3例CR、2例部分缓解（PR），ORR为71.4％。Ding等[46]报道了一项脐血来源MSC（hUCB-MSC）治疗激素耐药aGVHD的回顾性研究，研究纳入54例儿童及成人患者，中位输注次数为2（1～15）次，中位输注剂量为2.54×10^6^/kg，治疗28天ORR达59.3％；治疗第28 d达到缓解是生存的独立预后因素［总生存（OS），*P*<0.001；无进展生存（PFS），*P*<0.001；无GVHD/无复发生存（GRFS），*P*＝0.001］，而成年患者长期预后不佳（OS，*P*<0.001；PFS，*P*<0.001；GRFS，*P*＝0.003）。此外，其他组织来源的MSC的Ⅰ期临床研究也展现出良好疗效及安全性[47]–[48]。上述研究大多为回顾性研究或小样本量单臂研究，证据级别较低，而随机对照研究则因存在基础二线治疗选择，影响MSC疗效评价。

Kebriaei等[5]在2020年报道了一项Ⅲ期、双盲、随机研究。该研究共入组260例激素耐药的Ⅱ～Ⅳ度aGVHD患者，意向性治疗人群（ITT）中接受Remestemcel-L治疗的患者持久CR（CR持续28 d）率未能优于安慰剂对照组（35％对30％，*P*＝0.42）[5]。但后续分析发现，特定亚组人群如儿童患者、肝脏受累患者和明尼苏达高危患者亚群接受MSC治疗的ORR显著高于安慰剂。值得注意的是，该研究入组患者的干细胞来源（外周血来源占78％）、患者年龄（6个月至70岁，成人占84％）、GVHD预防方案（他克莫司53％、CsA 44％、MMF 33％）和基础二线治疗方案比例［兔抗人胸腺细胞免疫球蛋白（rATG）22％、MMF 17％、英夫利西单抗18％］存在异质性，可能对MSC疗效评估造成影响。

Zhao等[49]报告了一项开放标签、Ⅲ期、随机试验，结果显示骨髓来源的MSC联合巴利昔单抗和钙调蛋白抑制剂治疗激素耐药aGVHD患者的第28天ORR显著高于对照组（82.8％对70.7％，*P*＝0.043）；该试验研究组患者中位年龄28（16～59）岁，其中成人占85％；GVHD预防方案以CsA联合甲氨蝶呤（MTX）为基础，57.6％患者加用rATG；Ⅲ～Ⅳ度aGVHD占63.6％。Fu等[50]进行了一项开放标签、Ⅲ期、随机试验，结果显示接受脐带来源MSC联合巴利昔单抗治疗的成人激素耐药患者第28天CR率高于巴利昔单抗单药治疗患者（83.1％对55.4％，*P*＝0.001）；单药治疗组患者拥有更低的CD34^+^细胞数（*P*＝0.065），而两组患者的基线数据和GVHD分级无显著差异，Ⅲ～Ⅳ度aGVHD患者约占50％。上述两项研究虽然均为随机对照研究，但存在非盲和非安慰剂对照试验设计的局限性，可能会给研究结果带来偏倚。

Jiang等[6]报告了一项多中心、随机双盲、安慰剂对照的关于探索脐带来源MSC产品（hUC-MSC PLEB 001）联合除芦可替尼之外的二线药物（85％为巴利昔单抗）治疗Ⅱ度以上激素耐药aGVHD疗效和安全性的Ⅱ期临床研究结果，MSC组患者的中位缓解时间为14（5～22）d；ITT队列中，接受MSC治疗患者的第28天ORR高于对照组但差异无统计学意义（60％对50％，*P*＝0.375），MSC组中重度慢性GVHD的发生率低于对照组（13.8％对39.8％，*P*＝0.067）；但在8次及以上药物输注（治疗4周及以上）的患者中，MSC组第28天ORR显著高于对照组（66.7％对33.3％，*P*＝0.031）。此项研究中值得注意的发现有2点：MSC的中位起效时间为14（5～22）d，较芦可替尼［3（1～7）d］和巴利昔单抗［11（2～56）d］起效时间长；患者接受MSC治疗第56天的ORR为50％，其从第28天到第56天的疗效衰减幅度远小于REACH2研究中的芦可替尼ORR（第28天，62.3％；第56天，39.6％），提示脐带来源的MSC的临床作用特点为起效稍缓而疗效持久。

（二）MSC治疗激素耐药aGVHD的不同靶器官效果

Niu等[51]报道脐带来源的MSC治疗Ⅲ～Ⅳ度激素耐药的肠道aGVHD患者的长期随访结果，研究在激素和CsA基础治疗上应用脐带MSC治疗aGVHD中最为严重的类型即累及下消化道的Ⅲ～Ⅳ度患者，86例患者中位年龄为27.5（11～54）岁，均采用CsA联合MTX为基础的GVHD预防性用药，其中合并肝脏受累的患者占21％；治疗第28天的ORR为52.3％，合并肝脏受累的患者ORR为22.2％；第100天OS率为43.7％；中位随访108（61～159）个月后，仅有10例（11.6％）患者在随访结束时仍存活。Bukauskas等[52]同样报道了骨髓MSC治疗经活检证实的Ⅲ～Ⅳ度激素耐药的aGVHD患者的研究结果。此项研究纳入57例患者，中位年龄为55（19～71）岁，93％患者肠道受累，49％患者皮肤受累，25％患者肝脏受累，其中肠道合并其他器官受累占56％；患者总体治疗第28天的ORR为42％，肠道受累患者的ORR为38％；总体1年OS率为27％，而肠道受累患者的OS率为17％；MSC治疗14 d或28 d获得缓解的患者OS率显著高于未能获得缓解的患者（52％对7％，*P*<0.001；54％对5％，*P*<0.001）。Khan等[53]对已发表的随机对照研究进行荟萃分析，遵循严格的纳排标准，最终纳入Kebriaei等[5]、Jiang等[6]、Zhao等[49]、Fu等[50]的4项研究，共650例激素耐药aGVHD患者，其中514例患者肠道受累，310例患者肝脏受累，485例患者皮肤受累；分析结果提示MSC治疗皮肤和肠道受累患者疗效更加显著，而对于肝脏受累患者，MSC疗效不佳。

（三）MSC治疗aGVHD的疗效影响因素

影响MSC治疗aGVHD疗效的因素主要涉及患者因素和MSC制备及回输因素。其中，患者因素主要包括年龄、aGVHD分级和受累器官。在Kebriaei等[5]的研究中总体结果提示儿童患者的疗效可能优于成人患者；然而该研究只有13例患儿被随机分入了对照组，其中3例获客观缓解，而随机分入对照组的成人患者的ORR达60％，例数较少无法说明结论。在Temcell的回顾性研究中，三个年龄组（<18岁、18至49岁和≥50岁）的ORR无显著差异[4]。因此，没有充分证据证明MSC在儿童患者中的疗效高于成人患者。Chen等[54]的荟萃分析结果提示，MSC治疗激素难治性aGVHD，皮肤受累患者疗效优于胃肠道或肝脏受累者；Niu等[51]的研究表明，仅肠道受累患者疗效优于合并肝脏受累患者。

MSC输注剂量及应用时间对疗效的影响尚有争议。在前瞻性研究中，Remestemcel-L和Temcell以2×10^6^/kg的剂量每周输注2次，连续输注4周，而后每周输注1次，持续4周[4]–[5],[44],[55]。对日本Temcell真实世界数据的分析表明[4]，61％的患者接受了≤8次输注，39％的患者接受了9～12次输注；分别有50％和90％的患者（占OR患者比例）在接受Temcell治疗的第15天和第28天获得OR。以上数据表明对于大部分患者，8次MSC输注已足以达到预期疗效。国内部分MSC研究以1×10^6^/kg的剂量每周输注1次，连续4周，如第28天评估为PR则继续巩固4周[49]–[50]。Jiang等[6]的研究以1×10^6^/kg的剂量每周输注2次，如第28天评估为CR则停药，评估为PR则继续巩固4周。该研究发现共输注8次的患者亚群疗效显著优于对照组。

四、总结与展望

aGVHD的二线治疗用药机制已经从深度免疫抑制向炎症抑制的同时进行损伤修复、免疫调节方向发展。MSC作为兼备免疫调节和损伤修复作用的细胞群体，在血液病治疗、造血干细胞移植领域的应用值得进一步探索。MSC以激素耐药aGVHD为适应证在国内上市同样推动了其他免疫损伤类疾病的治疗进展。但MSC适应证的扩充还需要证据等级高、研究设计完备的随机对照试验作支撑和依据。此外，作为器官细胞的基质干细胞，MSC或许可以成为临床靶向治疗、基因治疗的载体，发挥更为广泛的临床效应。
